# From immature to mature epithelium: unveiling structural dynamics and transcriptional programs in rainbow trout intestinal barrier

**DOI:** 10.1186/s13567-026-01766-2

**Published:** 2026-09-15

**Authors:** Andrea Talamilla-Espinoza, Francisca Vera-Tamargo, Mario Caruffo, Miltha Hidalgo, Daniela Ortiz, Leonardo Rodríguez, Dinka Mandakovic, Ana Riveros, Omar Porras, Rodrigo Pulgar

**Affiliations:** 1 Genomics and Genetics of Biological Interactions Laboratory, Institute of Nutrition and Food Technology (INTA), University of Chile, El Líbano 5524, 7830490 Santiago, Chile; 2 Center for Research and Innovation in Aquaculture (CRIA), University of Chile, Santiago, Chile; 3 Laboratory for Research in Functional Nutrition, Institute of Nutrition and Food Technology (INTA), University of Chile, El Líbano 5524, 7830490 Santiago, Chile; 4 Molecular Mechanisms of Disease Laboratory, Center for Integrative Biology, Universidad Mayor, Camino La Pirámide 5750, 8580745 Santiago, Chile; 5 GEMA Center for Genomics, Ecology and Environment, Universidad Mayor, Camino La Pirámide 5750, 8580745 Santiago, Chile; 6 Laboratorio de Nanobiotecnología y Nanotoxicología, Departamento de Química Farmacológica y Toxicológica, Facultad de Ciencias Químicas y Farmacéuticas, Universidad de Chile, Sergio Livingston 1007, 8380492 Santiago, Chile; 7 Programa de Doctorado en Ciencias Silvoagropecuarias y Veterinarias, Campus Sur Universidad de Chile, Santa Rosa 11315, 8820808, Santiago, Chile

**Keywords:** RTgutGC cell line, in vitro intestinal barrier model, transcriptomic profiling, epithelial maturation, rainbow trout (*Oncorhynchus mykiss*), gut health

## Abstract

The intestinal epithelium is crucial for nutrient absorption, immune defense, and barrier function in farmed fish. However, the molecular mechanisms underlying its development and maturation in salmonids remain poorly characterized, hindering our ability to address pervasive gut health challenges in aquaculture. In this study, we use the RTgutGC cell line to implement an epithelial maturation model with the aim of characterizing the global transcriptional program in rainbow trout (*Oncorhynchus mykiss*). We evaluated in vitro culture conditions to generate a polarized epithelial barrier with high transepithelial electrical resistance (TEER = 75.8 Ω × cm^2^), low permeability (6.2 × 10^–6^ cm/s), and well-defined apical specializations, including microvilli-like structures and clusters of these structures (brush border). Comparative transcriptomic profiling between immature (7 days post-seeding, dps) and mature (28 dps) epithelia revealed 3,817 differentially expressed genes (DEGs). Functional enrichment analysis showed that maturation was characterized by the downregulation of proliferative and ribosomal pathways and the concerted upregulation of processes critical for barrier function, including transmembrane transport, proteolysis, cell adhesion, extracellular matrix organization, and tight junction assembly. We identified a core set of 60 genes indicators of epithelial maturation, encompassing solute transporters (*slc26a6, slc43a2*), tight junction proteins (*tjp1, cldn1, cldn3, cldn5, cldn18, among others*), and structural components essential for microvilli formation and polarization (*cdhr5b, pard6a*). By integrating ultrastructural, functional, and transcriptomic data, this study established a framework for future mechanistic investigations into gut development and maturation in vitro. This set of mature epithelium indicators has diverse applications, such as the design of nutritional and pharmacological interventions to improve gut health and resilience in farmed fish.

**Supplementary Information:**

The online version contains supplementary material available at https://doi.org/10.1186/s13567-026-01766-2.

## Introduction

Aquaculture has emerged as a pivotal contributor to global food security, projected to supply over 60% of aquatic food consumption by 2032 [1]. However, the sustainable growth of this sector faces challenges, including recurrent outbreaks of infectious diseases and shortages of dietary ingredients, such as fishmeal and fish oil [2, 3]. The intestinal epithelium plays a fundamental role in addressing these challenges, contributing to the performance of animal production by mediating crucial physiological processes: efficient nutrient absorption, robust immune responses, and selective barrier function against pathogens [4, 5]. These interconnected functions are facilitated by polarized enterocytes equipped with specialized apical structures, including microvilli that expand the absorptive surface area and tightly regulated junctional complexes that maintain epithelial integrity [6, 7]. While intestinal physiology has been extensively characterized in mammals, its regulation in teleost species remains poorly understood [8], and there are still gaps in understanding the structural and transcriptional programs driving epithelial development and maturation.

During epithelial differentiation and maturation, enterocytes develop a sophisticated polarized architecture comprising three distinct functional domains: the apical domain extends the microvilli into the intestinal lumen, the lateral domain establishes intercellular junctions to maintain epithelial integrity and intercellular communication, and the basolateral domain mediates attachment to the underlying basement membrane [6, 9, 10]. This spatial organization depends on highly regulated trafficking pathways that precisely localize membrane proteins, signaling receptors, and extracellular matrix components to their respective domains [11]. Although the core polarization machinery is evolutionarily conserved, recent studies reveal interspecies variation in its regulatory mechanisms, particularly in the timing and expression patterns of polarity factors [7, 12]. This variability underscores a critical knowledge gap in salmonids, where reliance on zebrafish models and mammalian biomarkers fails to capture species-specific features of intestinal epithelial development, potentially limiting our ability to address gut health challenges in these commercially important fish. This is particularly important for salmonids, which are naturally strict carnivores; however, they are raised on mixed diets of animal and plant origin, generating greater interest in assessments of these ingredients on gut health [13, 14].The use of in vitro approaches, such as well-characterized cell lines, could advance research on fundamental digestive functions and the effects of functional feed ingredients on intestinal immunity, barrier function, and digestion in fish [15]. In this context, the RTgutGC cell line, derived from rainbow trout (*Oncorhynchus mykiss*) distal intestine [16], has emerged as a promising model for addressing these issues. These cells form polarized monolayers on permeable supports, creating a two-compartment intestinal barrier system (bicameral chambers) that mimics the gut lumen (apical side) and bloodstream (basolateral side) [17]. Given that key functional properties, such as nutrient absorption, permeability regulation, intercellular junction formation, and enzymatic activity, have been described in epithelia formed by the RTgutGC cell line, and that its integrity as a barrier can be assessed by transepithelial electrical resistance (TEER) [15, 18, 19], this cell line represents a useful and physiologically relevant model comparable to the human Caco-2 cell line. This polarized cell line on permeable supports has been applied for investigating barrier function and ion/molecule transport across the intestinal epithelium [17, 18, 20–23]; characterization of the barrier and epithelial differentiation [19]; response to exposure to bile and nutritional deprivation [24, 25]; cytotoxicity [26–29] and barrier function and intestinal immunity [15, 30–36]. However, to our knowledge, it has not been used as a model to study the development and maturation of the intestinal epithelium in trout, at the level of structural and transcriptional programs.

To address this gap, we utilized the established RTgutGC cell line grown on permeable supports to generate polarized monolayers that partially recapitulate native epithelial architecture. We complement a follow-up of barrier formation and functional analysis, specifically TEER measurements, permeability assays, and ultrastructural characterization, with the first transcriptome-wide profiling of intestinal epithelial maturation in salmonids. This comprehensive approach enabled a comparison of global gene expression patterns between immature and mature epithelial in vitro cultures.

## Materials and methods

### RTgutGC cell line culture conditions.

The rainbow trout intestinal epithelial cell line RTgutGC [16] was routinely cultured at 18 °C under normal air atmosphere in Leibovitz’s medium containing 900 mg/L galactose, 10 mg/L phenol red, 300 mg/L L-glutamine, 550 mg/L sodium pyruvate (L-15, Gibco, #41300-039), 40 μM 2-mercaptoethanol (Sigma-Aldrich, #21985-023), 10 μg/mL ciprofloxacin (Sigma-Aldrich, #17850) for the control of *Mycoplasma sp*, and supplemented with 10% heat-inactivated fetal bovine serum (FBS; Sigma-Aldrich, #F9665). For experiments requiring polarized growth, cells were seeded onto 0.4-μm pore polycarbonate (PC; Corning, #3413) or polyester (PET; Corning, #3460) permeable supports (Transwell^®^ inserts). Prior to cell seeding, inserts were coated with Attachment Factor Protein (Gibco, #S006100) for 1 h; the coating solution was then aspirated, and cells suspended in culture medium were added to the treated inserts. Two insert sizes were employed: translucent 0.33 cm^2^ inserts (PC; 24-well plates) for standardizing growth conditions and transparent 1.2 cm^2^ inserts (PET; 12-well plates) to better monitor functional assays and obtain a larger cell biomass for RNA extraction for gene expression assays. In order to optimize the culture conditions for the RTgutGC cell line, we conducted an evaluation of two key parameters: initial cell seeding density and FBS supplementation. The study compared two seeding densities (62,500 versus 125,000 cells/cm^2^) and examined two different medium formulations: the L-15 medium described above supplemented with either 15% or 20% FBS. RTgutGC cells were transferred from Luis Mercado's laboratory (Pontificia Universidad Católica de Valparaíso, Chile) in passages 90, and experiments were performed between passages 95 and 115, similar to what has been described in other studies [19]. The apical and basolateral compartments received 300 μL/1 mL (PC; 0.33 cm^2^ inserts) or 1 mL/1.7 mL (PET; 1.2 cm^2^ inserts) of medium, respectively. Cells were maintained under these conditions for up to 28 dps, with weekly medium replacement to ensure nutrient replenishment.

The integrity and functionality of the resulting epithelium were quantitatively assessed through two complementary approaches: measurement of transepithelial electrical resistance (TEER) to evaluate formation and integrity of the barrier, and permeability assays using Lucifer Yellow (0.4 kDa) (LY; Invitrogen, #L453) to assess paracellular transport.

### Integrity and functionality of the intestinal epithelial barrier

Barrier formation was evaluated by monitoring transepithelial electrical resistance (TEER) using an epithelial voltmeter (EVOM2, World Precision Instruments) [37]. For each insert, the resistance (R) measurements of three different areas were averaged. Two inserts were used for four independent assays (n = 8). TEER were calculated with the following formula:

$$TEER \left(\Omega \times {cm}^{2}\right)=\left(R \left(sample\right)-R \left(blank\right)\right)\times insert growth area$$

For functional assessment, paracellular permeability was determined using Lucifer Yellow (LY) as previously described [18, 19, 24, 38, 39]. Briefly, both apical and basolateral compartments were washed with 1 × PBS (Gibco, #10,010,023) after medium removal. Subsequently, 200 μL of 1 mM LY solution (dissolved in PBS) was added to the apical chamber, while 500 μL of 1 × PBS was added to the basolateral chamber. Following a 1 (7 dps) or 2-h (28 dps) incubation in darkness, LY fluorescence in the basolateral compartment was measured (λex = 428 nm, λem = 536 nm) using a microplate reader (Tecan infinite M200). Apparent permeability (Papp) coefficients were calculated with the following formula:

$$Papp =\frac{VB}{(A\times CA0)\times (\frac{\Delta CB}{\Delta t})}$$

Papp: apparent permeability. VB: volume in the basolateral compartment. A: insert growth area. CA0: initial concentration in the apical compartment. ∆CB/∆t: change in concentration over time in the basolateral compartment.

### Epithelial polarization and structural characterization

Once the culture conditions that presented a higher TEER and a lower paracellular permeability to LY were selected, the characterization of the ultrastructure, polarity and transcriptional profile associated with the maturation and development of the epithelium in vitro was continued. To confirm cell polarization in the mature epithelium, immunofluorescence assays were performed to detect the apical tight junction protein ZO-1. For the evaluation of ZO-1, cells cultured for 7 dps and 28 dps on Transwell^®^ inserts were fixed with 4% paraformaldehyde (Sigma-Aldrich, #158127) for 10 min, permeabilized with 0.1% saponin (Chemcruz, #280079A) for 15 min, and blocked with 1% bovine serum albumin (BSA; Genesee scientific, #25–429) for 45 min. They were then incubated overnight at 4 °C with a polyclonal anti-ZO-1 rabbit primary antibody (1:200) (Thermo Fisher Scientific, #617300) [27], followed by incubation with a secondary goat anti-rabbit IgG Alexa Fluor™ 488-conjugated antibody (1:500) (Thermo Fisher Scientific, #A-11008) for 2 h and DAPI nuclear staining (Invitrogen, #D1306) for 10 min. Images were acquired using a Nikon C2 + confocal microscope. For 3D reconstruction, a survey of the full height of the cell monolayer was performed. Z-stacks were acquired and subsequently analyzed using Imaris software (Bitplane, v. 7.7) (9 slices to 1.5 μm step size for 7 dps and 20 slices to 1.5 μm step size for 28 dps).

To further characterize epithelial maturation and enterocyte-specific structures, scanning electron microscopy (SEM) was performed at 1, 7-, 14-, 21- and 28 dps on Transwell^®^ inserts. Cells cultured in the bicameral system were washed with PBS and fixed for 12 h using a 2% glutaraldehyde solution (Sigma-Aldrich, #G7651), 0.1 M sucrose, and 0.1 M sodium cacodylate (Sigma-Aldrich, #C0250). After six washes with 0.1 M sodium cacodylate, samples were post-fixed with 1% osmium tetroxide (Sigma-Aldrich, #75632) in 0.1 M sodium cacodylate for 1 h, followed by incubation with 1% tannic acid (Sigma-Aldrich, #T-0.125) for 1 h. Samples were then dehydrated in a graded ethanol series (nine steps) and dried with hexamethyldisilazane (Sigma-Aldrich, #440191). After air-drying for ≥ 12 h, samples were sputter-coated with 10 nm gold (Cressington 108 Auto Sputter Coater) before SEM imaging in an electron microscope (F50Field Electron and Ion Company). The measurement of structures in SEM was done manually using Fiji software. To assess the presence of partial or total multilayer formation, Z-stack confocal images of seven supports with mature epithelium (28 dps) were reviewed. To estimate the extent of stratification, nuclear overlap was evaluated. Images were processed using NIS-Viewer software (version 4.20, b972), and Z-stacks were rendered with depth-coding. Subsequently, a manual count was performed using Fiji software to calculate the proportion of overlapping nuclei relative to the total number of nuclei.

### RNA sequencing and transcriptomic analysis of intestinal epithelial maturation

Total RNA was extracted from three independent biological replicates of immature (7 dps) and mature (28 dps) RTgutGC cell epithelia cultured on Transwell® inserts, using TRI Reagent® (Sigma-Aldrich). RNA quantity and purity (260/280 nm absorbance ratio) were assessed using a NanoDrop 2000 Spectrophotometer (Thermo Fisher Scientific), while integrity was verified via RNA Integrity Number (RIN ≥ 8.5) on a 2200 TapeStation Instrument (Agilent Technologies). Only high-quality samples (260/280 ≥ 1.8; RIN ≥ 8.5) were processed further. For RNA-seq library preparation, mRNA was enriched using the Dynabeads™ mRNA Purification Kit (Invitrogen), followed by library construction with the MGIEasy RNA Library Prep Kit and MGIEasy Circularization Module V2.0 (MGI). Paired-end sequencing (2 × 150 bp) was performed on a DNBSEQ-G400 platform (MGI) with the DNBSEQ-G400RS High-throughput Sequencing Set, yielding 30.7- 46.5 million reads per sample. Raw FASTQ files were quality-checked with FastQC (v0.12.1) [40], and low-quality reads (Phred score < 30, length < 75 bp) were filtered out using Trim Galore (v0.6.10) [41]. High-quality reads were aligned to the *Oncorhynchus mykiss* reference genome (GCF_013265735.2, NCBI RefSeq assembly) and quantified using STAR (v2.5.2b) [42]. Raw counts were normalized via the Trimmed Mean of M-values (TMM) to correct for library size differences. Differential expression analysis was performed using limma (v3.62.2) [43], with differentially expressed genes (DEGs) by comparing mature (28 dps) to immature (7 dps) epithelia |log_2_FC|> 1 and adjusted *p*-value (FDR) < 0.05. Results were visualized using packages in R environment (version 4.4.2) for Multidimensional Scaling (MDS) (limma), heatmaps (ComplexHeatmap, v2.22.0), and volcano plots (EnhancedVolcano, v1.2.0). For functional annotation, DEGs were analyzed in Database for Annotation, Visualization, and Integrated Discovery (DAVID) for Gene Ontology (GO) enrichment (biological process, molecular function, cellular component) and KEGG pathway analysis. Enriched pathways were selected considering the adjusted *p*-value (Benjamini-Hochberg < 0.05), gene count and fold enrichment cutoff. The results were visualized in a dotplot using the ggplot2 package (v3.5.2) in R. RNA-seq raw data were submitted to NCBI Sequence Read Archive (SRA), accession number: SRP682370.

### Statistical analysis

Statistical analyses and data visualization were conducted using GraphPad Prism (version 8.0.1) and R environment (version 4.4.2). Evaluation of data normality was performed using the Shapiro–Wilk test. A one-way ANOVA was used as the main statistical test, followed by a Tukey post hoc analysis to evaluate the effect of the number of cells seeded and the concentration of FBS on TEER and permeability. A Student's *t*-test was performed to evaluate differences in TEER and epithelial permeability based on the material of the permeable supports (PC or PET) and cell culture time (7 or 28 dps). Student's *t*-test was also used to compare the effect of cell culture time on the length and width of epithelial microvilli-like. Data were presented as the mean ± standard deviation (SD). Values of *p*-value < 0.05 were considered statistically significant.

## Results

### Integrity and functionality of the intestinal epithelial barrier

**Figure 1:**
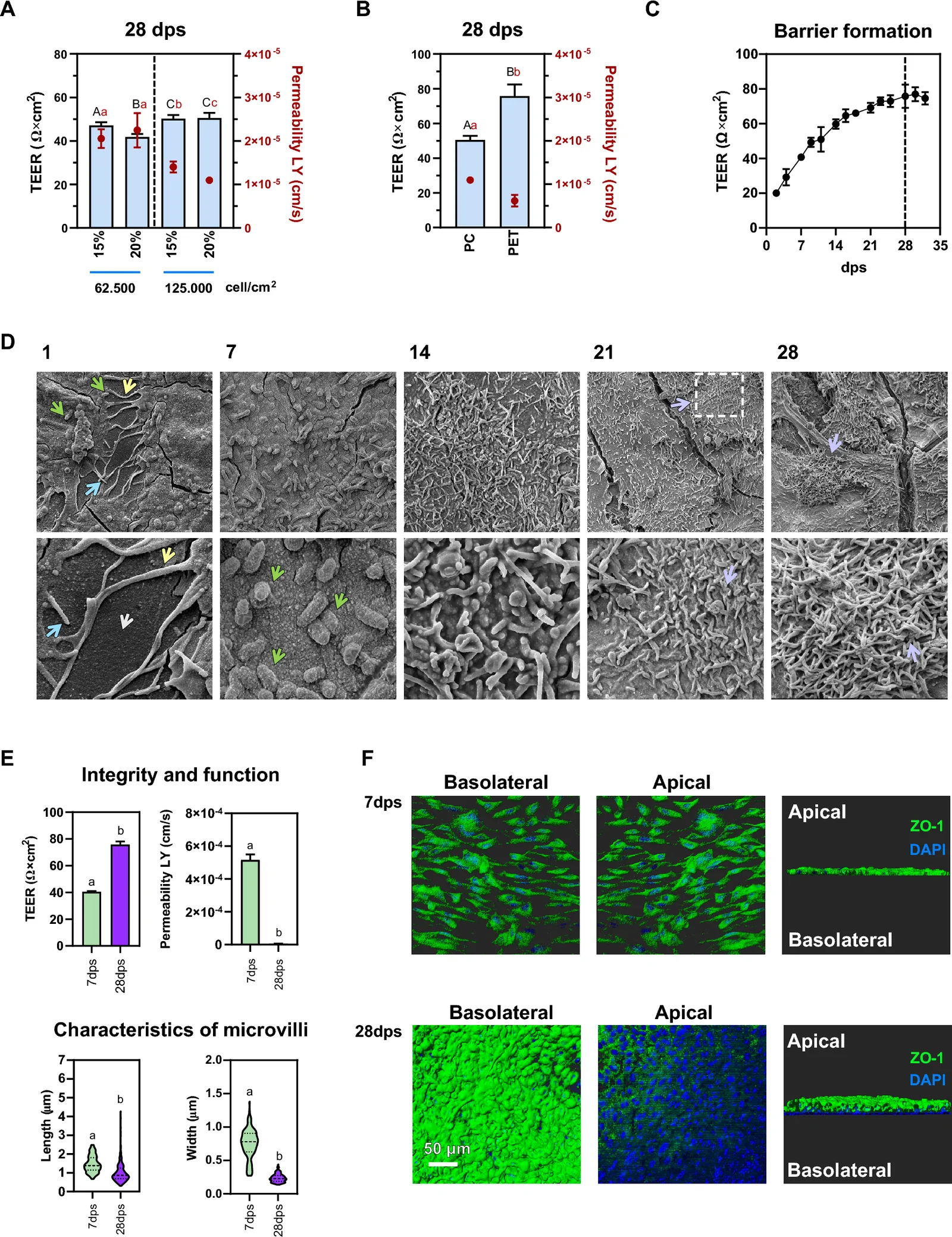
**Evaluation of culture conditions and characterization of a polarized RTgutGC intestinal epithelium in vitro. A** Impact of seeding density and fetal bovine serum (FBS) concentration on epithelial integrity, measured by transepithelial electrical resistance (TEER) (*n* = 6), and barrier function, assessed by lucifer yellow (LY) permeability (*n* = 4). **B** Comparison of permeable supports material (polycarbonate, PC; polyester, PET) on epithelial integrity and function (*n* = 6). **C** Time course of epithelial barrier formation monitored by TEER over 32 days post-seeding (dps). Cells were seeded at 125 000 cells/cm^2^ on PET membranes and cultured in L-15 medium with 20% FBS. **D** Ultrastructural maturation of the epithelium analyzed by scanning electron microscopy (SEM) at the indicated time points. Representative images show key developmental structures: filopodia (light blue arrows), tunneling nanotube-like structures (yellow arrows), exposed support surface (white arrow), microvilli-like (green arrows), and microvilli-like clusters (light purple arrows) top panel: 8000 × magnification; bottom panel: 30 000 × magnification of the same field of view. **E** Top panel, Comparative analysis of the integrity and function of immature and mature epithelium (*n* = 4). Bottom panel**,** quantification of the length and width of microvilli-like in immature epithelium (7 dps) (length *n* = 188, width *n* = 111) and in mature epithelium (28 dps) (length *n* = 195, width *n* = 109). For quantitative analysis, manual measurements were performed in Fiji software and significance was determined using Student’s *t*-test in GraphPad Prism. **F** Apical polarity assessment at 7 and 28 dps by immunofluorescence staining for the apical tight junction protein ZO-1 (green) and nuclei (DAPI, blue) 3D reconstruction was generated from a confocal Z-stack using Imaris software (Bitplane, v 77). *p*-value < 0.05 were considered statistically significant.

To establish optimal culture conditions for an intact RTgutGC epithelium in a bicameral system, we evaluated key variables from prior studies. All cultures were maintained in Leibovitz's L-15 medium (L-15) while we investigated the effects of seeding density, fetal bovine serum (FBS) concentration, and support membrane material over 28 dps. Epithelial integrity was assessed by measuring transepithelial electrical resistance (TEER) and permeability to Lucifer Yellow. As shown in Figure 1A, the optimal conditions in PC inserts, yielding the highest TEER (ANOVA: F (3, 20) = 29.82, *p*-value =  < 0.0001) and lowest permeability (ANOVA: F (3, 60) = 78.28, *p*-value =  < 0.0001), were a seeding density of 125 000 cells/cm^2^ and L-15 medium supplemented with 20% FBS. Under these defined conditions, a subsequent comparison of permeable supports materials revealed that epithelial integrity was higher on polyester (PET) than on polycarbonate (PC) membranes (Figure 1B) (TEER t-test: t = 8.694, df = 12, *p*-value = 0.0384; permeability t-test: t = 12.90, df = 60, *p*-value = 0.0034). Based on this result, we monitored TEER over 32 dps to assess barrier formation and integrity. As shown in Figure 1C, the TEER increased over time, reaching a clear plateau at 28 dps (75.8 Ω × cm^2^). At this point, barrier functionality was confirmed by a lucifer yellow (LY) permeability assay, which reached a value of 6.2 × 10^–6^ cm/s (Figure 1B).

### Maturation and polarity of the intestinal epithelial barrier

Once the growth conditions necessary for the formation of the epithelial barrier in terms of functionality and integrity were selected, formation of characteristic structures during the maturation and polarization of the epithelium were evaluated. To characterize enterocyte maturation and ultrastructural development, we analyzed the epithelium by scanning electron microscopy (SEM). The results indicate that on day 1 dps, cells mainly exhibited lateral projections resembling-tunneling nanotubes and filopodia along with few microvilli-like structures on the apical surface (Figure 1D). The monolayer was initially discontinuous correlating with a low baseline transepithelial electrical resistance (TEER) value (20 Ω × cm^2^). At 7 dps, we observed that cells exhibited apical projections, with abundant microvilli-like structures distributed homogeneously across the cell surface. These were exclusively individual structures, with no evidence of grouped arrangements. Since epithelial maturation is characterized by the appearance of microvilli with their subsequent reorganization and clustering, 7 dps interval was chosen to represent an immature developmental state. This stage was characterized by the appearance of abundant microvilli-like structures on the surface without evidence of clustering, and with a low barrier function compared to the 28 dps (permeability of LY = 5.3 × 10^–4^ cm/s vs 6.2 × 10^–6^ cm/s at 28 dps), correlating with a lower TEER regarding the 28 dps (40.7 Ω × cm^2^ vs 75.8 Ω × cm^2^ at 28 dps) (Figure 1E) (permeability t-test: t = 15.56, df = 6, *p*-value < 0.0001; TEER t-test: t = 14.51, df = 14, *p*-value = 0.0004). From 14 dps onwards, the epithelium exhibited increasing structural complexity, marked by the progressive clustering of microvilli-like structures (Figure 1D). By 28 dps, TEER values had plateaued at 75.8 Ω × cm^2^, permeability was minimized and microvilli-like clustering was most complex and pronounced. Hence, based on these criteria, we selected the 28 dps time point as representative of a mature epithelium.

The comparative analysis of mature (28 dps) and immature (7 dps) epithelium, showed differences in microvilli-like structures morphology (Figure 1E). At 7 dps, microvilli-like clusters were significantly longer and wider compared to 28 dps. Specifically, mean length decreased from 1.47 ± 0.44 μm at 7 dps to 1.03 ± 0.58 μm at 28 dps (t = 8.32, df = 384, *p*-value = 0.0001), while mean width decreased from 0.75 ± 0.22 μm at 7 dps to 0.24 ± 0.06 μm at 28 dps (t = 23.43, df = 219, *p*-value < 0.0001). In addition, at 7 dps, Zonula Occludens-1 (ZO-1) showed a distribution from both the apical and basolateral views in immunofluorescence assays. In contrast, at 28 dps, increased epithelial complexity (higher epithelium) accompanied by polarization was observed, as confirmed by the predominantly apical localization of ZO-1 (Figure 1F). These structural findings were consistent with the higher TEER and lower permeability observed at 28 dps (Figure 1E). In addition, to determine whether the use of 20% FBS affected epithelial architecture, we performed a rigorous quantitative analysis of multiple confocal stacks. As shown in Additional file 1A-B, the predominant architecture at 28 dps is a continuous, polarized monolayer, comprising approximately 72.4% of the area. The remaining 27.6% consists of focal areas where a subset of cells forms a secondary layer, typically no more than a bilayer. Finally, SEM analysis also allowed us to observe a rosette-like apical structure on the epithelial surface (Additional file 2). These structures were heterogeneous in size and shape but widely distributed and present throughout the time course.

### Gene expression profile and transcriptional regulation of intestinal epithelial polarization and maturation in vitro

**Figure 2:**
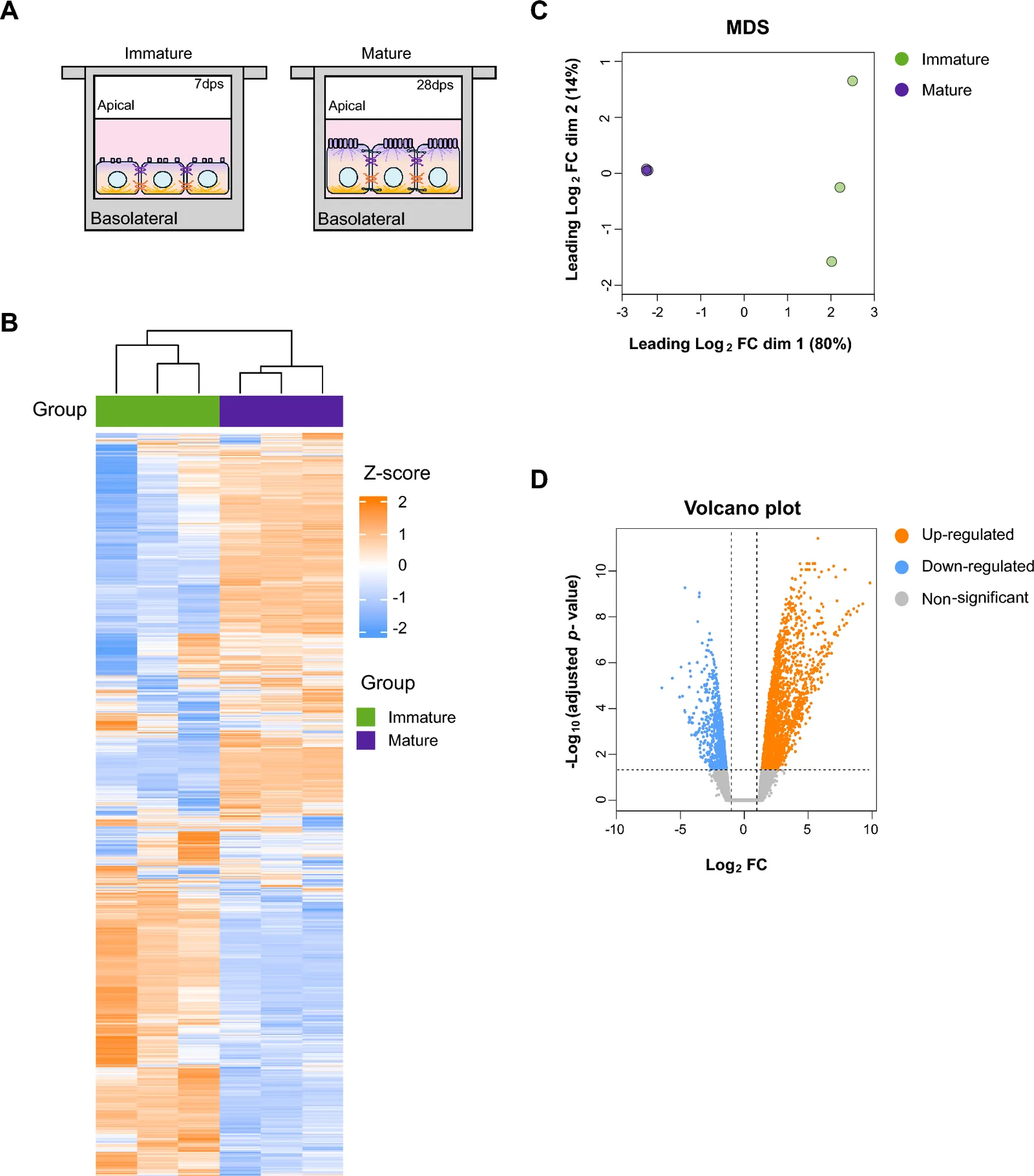
**Transcriptomic profiling of immature and mature RTgutGC epithelia. A** Schematic representation of experimental design, comparing immature (7 dps) and mature (28 dps) epithelial cultures. **B** Hierarchical clustering of gene expression profiles for biological replicates (*n* = 3) of immature and mature epithelia. The heatmap illustrates clear segregation of samples by developmental stage. **C** Multidimensional scaling (MDS) plot confirming distinct transcriptional profiles between immature and mature conditions. Each point represents a biological replicate. **D** Volcano plot of differentially expressed genes (DEGs). Genes with differential expression (adjusted *p*-value < 0.05 and |log_2_ fold change|> 1) are highlighted in orange (up-regulated, *n* = 2496) and light blue (down-regulated, n = 1321). Non-significant genes are shown in gray.

Based on the morphological and functional differences between immature (7 dps) and mature (28 dps) RTgutGC epithelia (Figure 2A), we undertook a comprehensive comparative transcriptomic-wide analysis via RNA sequencing (RNA-Seq). This approach was designed to elucidate the global gene expression patterns and key regulatory networks underlying epithelial maturation and functional polarization. To assess overall transcriptional differences, we first performed an unsupervised analysis of the gene expression data from biological replicates for each condition (immature and mature epithelium). Hierarchical clustering analysis revealed a clear segregation of samples, where replicates from the same developmental stage clustered together with high bootstrap support, while forming two distinct clades corresponding to the immature and mature states (Figure 2B). This pronounced segregation was further corroborated by multidimensional scaling (MDS) analysis, which showed a clear separation of sample groups along the primary coordinate (MDS1), with minimal intra-group variation (Figure 2C). Together, these analyses confirm that the developmental stage is the primary source of transcriptional variation in our system. Subsequently, we conducted a differential gene expression analysis to quantify these changes. Using a stringent threshold (adjusted *p*-value < 0.05 and |log_2_ fold change|> 1), we identified 3,817 differentially expressed genes (DEGs) in mature compared to immature epithelia, with 2496 up-regulated and 1321 down-regulated genes (Figure 2D; Additional file 3). Transcriptomic profiling revealed that maturation of the intestinal epithelium was marked by extensive functional specialization, evidenced by up-regulation of genes encoding proteins critical for nutrient absorption (e.g., aquaporins *aqp1* and *aqp10*, fatty acid transporter FABP, and multiple solute carriers), barrier integrity (claudins *cldn1*, *cldn3*, and *cldn15l1*, mucin *muc5ac*, and adhesion molecules such as cadherin *cdh6*, and digestive metabolism (amylase, dipeptidyl peptidase *dpp4*, and lipases). Concurrently, enhanced expression was observed for immune mediators (complement components C3 and C4, chemokine receptors), developmental signaling regulators (*dkk3b*, *grem1b*), and terminal differentiation markers, accompanied by down-regulation of cell cycle promoters. This expression profile indicates a transition from a proliferative, immature state to a functionally mature epithelium specialized for absorption, defense, and mucosal homeostasis. The breadth and magnitude of differential gene expression reflect a comprehensive reprogramming toward a polarized, functionally integrated epithelial phenotype.

**Figure 3:**
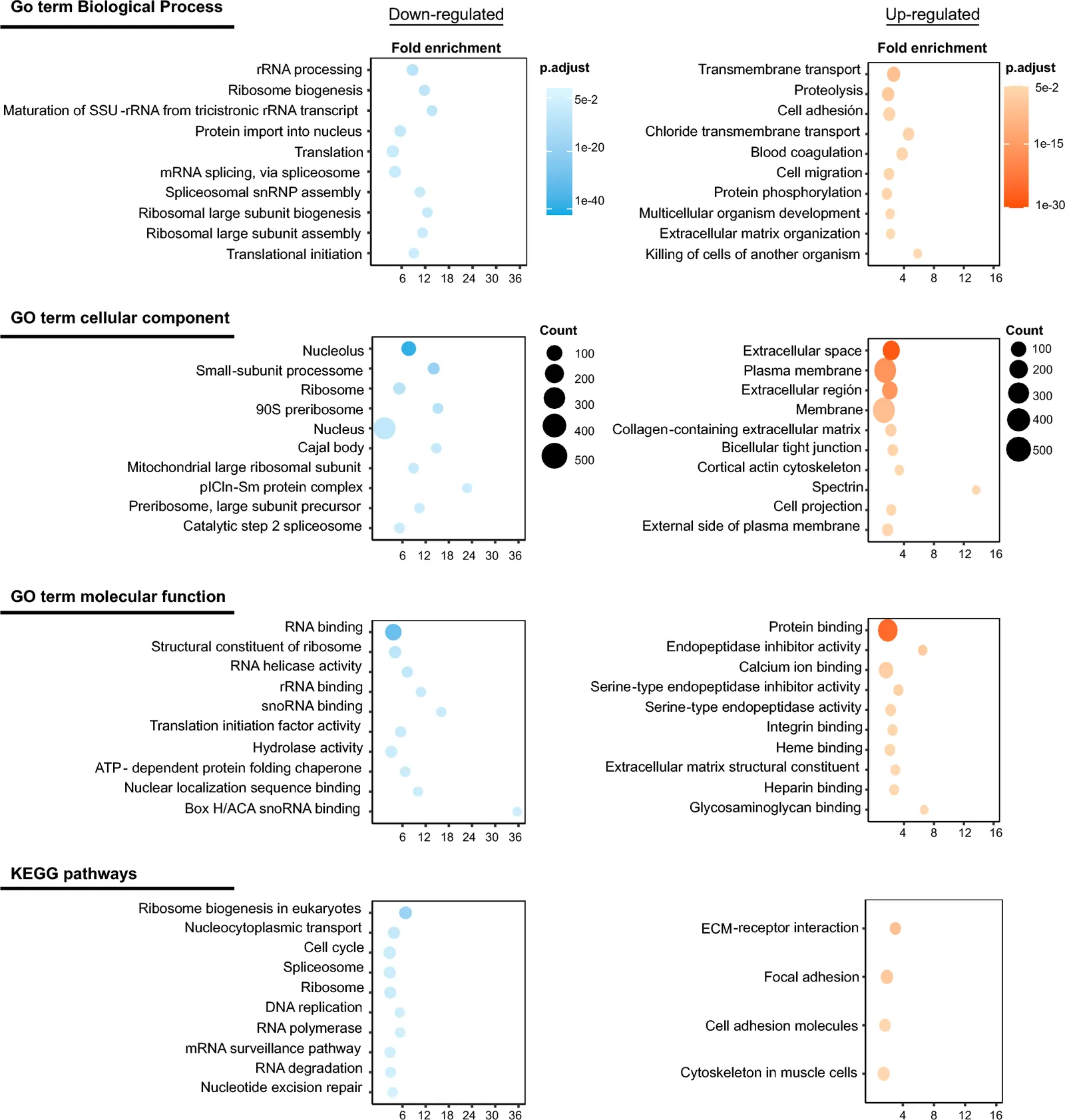
**Functional enrichment analysis Top 10 GO terms and metabolic pathways (KEGG) of differentially expressed genes modulated during epithelial maturation.** The left panel, in light blue, corresponds to pathways enriched for genes that were down-regulated in immature epithelium compared to mature epithelium. The right panel, in orange, corresponds to pathways enriched for genes that were up-regulated. Enriched pathways were selected considering the adjusted *p*-value (Benjamini-Hochberg < 0.05), gene count and fold enrichment cutoff, The results were visualized in a dotplot using the ggplot2 package (v3.5.2) in R.

To decipher the biological importance of the observed transcriptomic changes, we performed Gene Ontology (GO) and Kyoto Encyclopedia of Genes and Genomes (KEGG) pathway enrichment analyses on the down-regulated and up-regulated gene sets, separately (Additional file 3). Functional annotation of down-regulated genes revealed an enrichment of terms associated with highly proliferative and basal cellular functions. Biological processes were dominated by rRNA processing, ribosome biogenesis, maturation of Small Subunit Ribosomal RNA (SSU-rRNA), protein import into nucleus, and translation. These processes were supported by molecular functions such as RNA binding, structural constituent of ribosome, RNA helicase activity, rRNA binding, snoRNA binding, and translation initiation factor activity. Consistent with these terms, the predominant cellular components included the nucleolus, small-subunit processome, ribosome, 90S preribosome, and nucleus. KEGG pathway analysis complemented these findings, showing enrichment in pathways for ribosome biogenesis in eukaryotes, nucleocytoplasmic transport, cell cycle, spliceosome and ribosome (Figure 3, left). Together, these processes highlight the importance of cell division and the underlying regulation and production of transcripts and proteins in the development of immature epithelium. Conversely, analysis of up-regulated genes revealed a striking enrichment of terms related to epithelial maturation and specialization. Key biological processes included transmembrane transport, proteolysis, cell adhesion, chloride transmembrane transport and cell migration. The enriched molecular functions found were protein binding, endopeptidase inhibitor activity, calcium ion binding, serine-type endopeptidase inhibitor activity, integrin binding, and extracellular matrix structural constituent. Cellular component terms were strongly associated with extracellular space, plasma membrane, membrane, collagen-containing extracellular matrix, and bicellular tight junction. KEGG pathway analysis further demonstrated enrichment in Extracellular Matrix receptor (ECM-receptor) interaction, focal adhesion, cell adhesion molecules, and cytoskeleton in muscle cells (Figure 3, right). This coherent pattern of up regulation suggests a concerted role for these genes and processes in driving the polarization and functional maturation of the intestinal epithelium.

**Figure 4:**
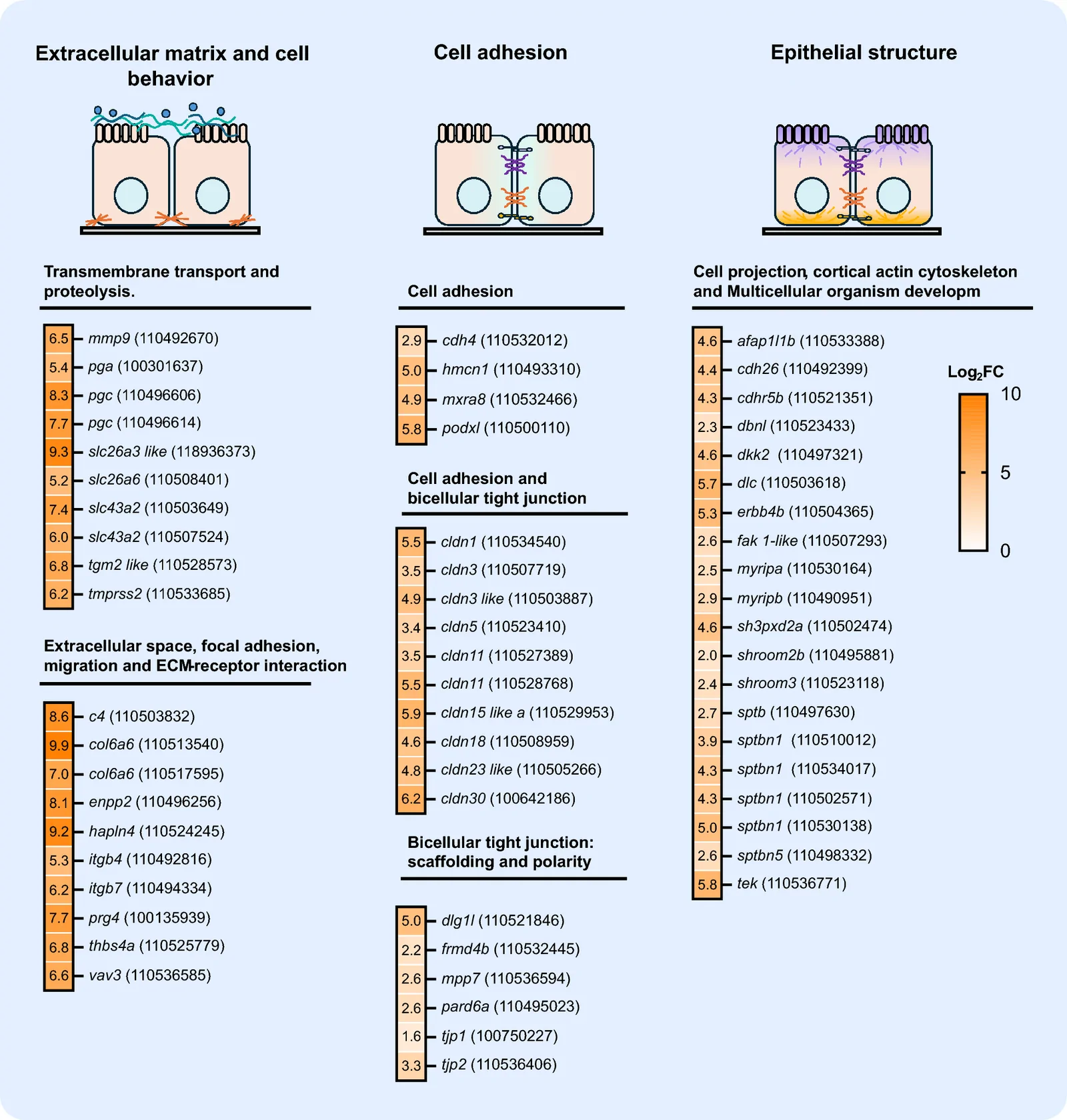
**Identification of indicators of polarization and maturation of the intestinal epithelium in vitro.** Selection of representative genes involved in intestinal epithelial polarization and maturation is shown, separated into three categories. This selection was based on enrichment analysis and is accompanied by a heatmap indicating the fold changes (log_2_FC) values in this diagram. The names reported correspond to the protein encoded by the gene, and the value in parentheses corresponds to its gene ID.

To identify putative indicators of epithelial polarization and maturation, we curated a set of 60 genes that were up-regulated in the mature epithelium relative to its immature state and enriched in relevant Gene Ontology terms or metabolic pathways. These candidate indicators were categorized into three core functional groups: (1) extracellular matrix and cell behavior, (2) cell adhesion, and (3) epithelial structure. Gene selection was performed by ranking genes within each enriched ontology or metabolic pathway based on the magnitude of their fold change. The results showed in the Figure 4 indicate that “the extracellular matrix and cell behavior” category comprises top-ranked genes from pathways including transmembrane transport (GO:0055085), proteolysis (GO:0006508), chloride transmembrane transport (GO:1902476), extracellular space (GO:0005615), cell migration (GO:0016477), focal adhesion (KEGG: omy04510), and ECM-receptor interaction (KEGG:omy04512). This group includes solute transporters such as the methionine and phenylalanine uniporter (*slc43a2*, 110503649) and the anion exchanger (*slc26a6*, 110508401), alongside proteolytic enzymes like gastricsin (*pgc*, 110496606) and transmembrane serine protease 2 (*tmprss2*,110533685). We also identified key structural and signaling components of the extracellular matrix, including collagen alpha-6(VI) chain (*col6a6*, 110513540), integrins beta-7 and beta-4 (*itgb7*,110494334 and *itgb4*, 110492816), thrombospondin-4a (*thbs4a*, 110525779), components of the immune system, such as complement c4 (*c4*, 110503832) and the signaling protein guanine nucleotide exchange factor (*vav3*, 110536585). “The cell adhesion” category is defined by genes enriched in pathways for cell adhesion (GO:0007155), bicellular tight junction (GO:0005923), and cell adhesion molecules (KEGG: omy04514). It is primarily represented by adhesion molecules such as podocalyxin (*podxl*, 110500110), cadherin-4 (*cdh4*, 110532012), and hemicentin-1 (*hmcn1*, 110493310). This group also features a wide array of genes that code tight junction proteins, including multiple claudins (*cldn*), as well as central scaffolding and polarity regulators like zonula occludens-1 and −2 (*tjp1*, 100750227 and *tjp2*,110536406), discs large homolog 1 (*dlg1l*, 110521846), and partitioning defective 6 homolog alpha (*pard6a*, 110495023). Finally, the “epithelial structure” category includes genes associated with cell projection (GO:0042995), cortical actin cytoskeleton (GO:0030864), and multicellular organism development (GO:0007275). This group features various spectrins (*sptb*, *sptbn*), cytoskeletal proteins that bind actin to regulate cell shape and facilitate intracellular trafficking, thereby promoting the formation of cellular projections like microvilli. Shroom3 (*shroom3*, 110523118), whose encoded protein regulates cellular projections by promoting the apical accumulation of F-actin and myosin II, is also included in this group. Together, these DEGs delineate the core molecular signature of a functional intestinal epithelium, providing not only critical insights into barrier formation but also a robust framework for assessing the maturity of in vitro piscine epithelial models.

## Discussion

In this study, we implemented an in vitro model of the rainbow trout intestinal epithelium using the RTgutGC cell line cultured in a two-compartment system to unveil the structural dynamics and transcriptional programs underlying epithelial maturation. While this model has been used in prior studies to investigate nutrient transport, barrier function, cytotoxicity, and immunity, a comprehensive characterization of its maturation process remains unprecedented.

To achieve a robust model, we maintained previously identified constant factors, such as Leibovitz's L-15 medium, and systematically defined key variables including seeding density, fetal bovine serum (FBS) concentration, and support membrane material over an extended (28 dps) period. This approach identified that cultures with a seeding density of 125 000 cells/cm^2^ on polyester (PET) membranes in L-15 medium supplemented with 20% FBS exhibited the highest transepithelial electrical resistance (TEER) and the lowest permeability values. Furthermore, the barrier integrity achieved under these specific conditions was maintained for up to 32 dps. Notably, the TEER value under these optimized conditions reached 75.8 Ω × cm^2^, exceeding the range of 25–40 Ω × cm^2^ previously reported for this cell line at comparable time points [15, 17, 18]. We attribute this enhancement primarily to the higher FBS concentration (20%) used here, compared to the 5–10% typically employed, a finding consistent with earlier growth optimization studies [16]. Nevertheless, this TEER remains markedly lower than values characteristic of mammalian intestinal models (e.g., > 300 Ω × cm^2^ in human Caco-2 cells) [44], underscoring inherent physiological differences between piscine and mammalian epithelial barriers. Furthermore, epithelial maturity is achieved at 28 dps in the salmonid RTgutGC model, compared to 21 dps in the human Caco-2 cell line [45]. This delayed maturation may be associated with the slower metabolic rates and reduced tissue biosynthesis characteristics of cold-water fish such as salmonids [46].

For our model, stabilization of transepithelial electrical resistance (TEER) served as the primary criterion for defining maturity. Accordingly, our data showed that this stabilization was not achieved by 21 days post-seeding (dps) but was reached at 28 dps. This supported the selection of this later timepoint as indicative of a mature epithelium, consistent with findings previously reported by other authors [15, 18]. However, the use of 20% FBS for 28 days might suggest the formation of a multilayered epithelium, resulting in high TEER values, a possibility that has also been discussed by other authors (e.g., Verdile et al. [47]). To address this, we performed a rigorous quantitative analysis of multiple confocal stacks, which revealed that, at 28 dps, the epithelium was predominantly a continuous and polarized monolayer. This monolayer accounted for approximately 72.4% of the analyzed area, while the remaining 27.6% corresponded to focal regions containing a secondary cell layer (Additional file 1A–B). Although a perfectly uniform monolayer would provide a more stringent simulation of an in vivo system, the combined assessment of our data, integrity (TEER stabilization) and functionality (permeability and specialized structures), robustly supports the conclusion that RTgutGC cells form a functional, mature, and predominantly monolayered intestinal barrier at 28 dps, making it a suitable model for our study. Furthermore, the maturity of the 28 dps epithelium is corroborated by the upregulation of genes encoding established functional markers of differentiated enterocytes in other rainbow trout intestinal models [19]. These include the sodium-glucose co-transporter 1 (*sglt1*110509885), leucine aminopeptidase (110536209), and alanine aminopeptidase (110493779).

We next evaluated epithelial polarization using immunofluorescence staining for the tight junction protein Zonula Occludens-1 (ZO-1) and nuclei (DAPI). At 28 dps, apical ZO-1 localization and nuclei preferentially localized in the basal region were observed, consistent with a columnar epithelium configuration, confirming the formation of a polarized epithelium. Subsequent scanning electron microscopy (SEM) analysis revealed a clear structural progression during epithelial development, with distinct early and late maturation characteristics. At the initial stage (1 dps), cells exhibited lateral projections resembling tunneling nanotubes and filopodia. Such membrane protrusions facilitate cytoplasmic transfer and environmental sensing, consistent with a migratory phenotype and a non-intact, permeable epithelium [48, 49]. During intermediate stages (7—21 dps), the epithelium developed increasing structural complexity, marked by a progressive rise in microvilli-like abundance and the emergence of clustered microvilli-like formations, an organization consolidated by 28 dps. As observed in human epithelial models, microvilli-like structures appeared stochastically rather than synchronously during differentiation [10] and the co-existence of nascent and mature microvilli-like structures indicated continuous remodeling during polarization. Enhanced microvilli-like structures clustering [10, 50] along with high-density zones and membrane folding in the mature RTgutGC epithelium, provided structural evidence of barrier maturation, aligning with apical ZO-1 localization.

In addition to microvilli-like, SEM analysis revealed an apical rosette-like structure of heterogeneous size that was observed at all times evaluated. Morphology led us to hypothesize that this was a basal body or transition zone of a primary cilium. However, its 1.56 µm diameter represented a sixfold increase over the standard diameter for these structures in human epithelia (0.25–0.30 µm), despite falling within the typical overall length range (1–9 µm) [51, 52]. The ubiquity of these structures across the epithelium highlights the need for a more detailed analysis of their nature.

Afterward, given the morphological and functional differences between immature (7 dps) and mature (28 dps) RTgutGC epithelia, we performed a comprehensive comparative transcriptomic analysis using RNA sequencing (RNA-Seq). Our results indicated that transcriptomic profiles clearly distinguished immature and mature epithelia, underscoring a fundamental molecular distinction between these developmental states. This transcriptional divergence is directly attributed to the extensive molecular reprogramming that characterizes epithelial maturation, which is quantitatively reflected in the identification of 3,817 differentially expressed genes (DEGs) between the two conditions. This finding is consistent with transcriptomic studies of human intestinal epithelial cells (hIECs) cells differentiated on permeable supports [53] and mouse small intestinal enterocytes [54], which also exhibit distinct transcriptional profiles based on their maturation stage.

Functional enrichment analysis revealed a dual transcriptional pattern consistent with the epithelial developmental state, reflecting both conserved mechanisms and teleost-specific adaptations. Genes down-regulated in the mature epithelium (or up-regulated in the immature epithelium) were enriched in processes related to cell proliferation, ribosome biogenesis, and RNA processing. This finding is consistent with established in vivo models of epithelial differentiation in humans and zebrafish, where intestinal progenitor cells proliferate in a niche before migrating and differentiating, a process that involves a well-documented down-regulation of proliferation signals and cell cycle machinery [9, 55]. Conversely, the up-regulation of genes in the mature epithelium marks the acquisition of specialized functions required for a polarized intestinal barrier, displaying both evolutionarily conserved and teleost-specific features. Enrichment analyses identified critical pathways and processes, including transmembrane transport, proteolysis, cell adhesion, extracellular matrix organization, tight junction assembly, focal adhesion, and cell projection, that collectively outline a molecular signature of active epithelial polarization. This underscores the fundamental role of matrix anchorage in establishing cell polarity [9], a mechanism also documented during Caco-2 maturation [56]. This functional signature aligns with in vivo zebrafish models, particularly in processes such as transmembrane transport, proteolysis, actin cytoskeleton organization, peptidase activity, and complement activation, reflecting core intestinal functions including digestion, nutrient absorption, and innate immunity [57]. However, whereas zebrafish models emphasize lipid and carbohydrate metabolism, our system showed elevated expression of amino acid transporters and proteolytic enzymes, a divergence likely driven by the carnivorous nature of salmonids [57, 58]. Additionally, solute transporters mediating chloride and bicarbonate exchange contribute to ion homeostasis, a trait vital for anadromous species such as salmonids to acclimate to seawater conditions [59].

Finally, based on the processes enriched in the mature epithelium, we identified some key molecular indicators of functional maturation, including solute transporters such as *slc26a6* (a chloride exchanger homologous to human SLC26A3) and *slc43a2* (an amino acid transporter), which reflect enhanced absorptive capacity and evolutionary conservation [60, 61], along with structural extracellular matrix components (*col6a6, itgb4, itgb7*) and proteases (*tmprss2*) indicative of active basement membrane remodeling critical for cell signaling and apicobasal polarity [9, 56]. Barrier formation was further evidenced by the up-regulation of the specific wide claudin profile (*cldn3*, *cldn25*) suggests a unique paracellular “permeability signature,” likely underlying the characteristically higher ionic permeability documented in teleost intestines [62, 63]. In addition, barrier formation was accompanied by the up-regulation of core tight junction proteins *tjp1* (ZO-1) and *tjp2* (ZO-2), mirroring their established role in mammalian models [64], along with the upregulation of conserved polarity regulators *dlg1l* (DLG1 homolog) and *pard6a* (PARD6A homolog), which confirmed the activation of canonical Par/aPKC pathways essential for epithelial maturation and polarization [6 , 65]. For its part, the assembly and dynamics of microvilli-like structures were supported by actin cytoskeletal regulators, including *afap1l1b*, *shrooms*, and *dbnl*, which facilitate structural organization and stability [65]. Functional homology in microvillar clustering across vertebrates was further indicated by the presence of *cdhr5* (protocadherin-24), a core component of the intermicrovillar adhesion complex (IMAC) [10, 50, 66]. Additionally, genes encoding protein tyrosine kinase receptors such as *erbb4b* and *tek* were identified, underscoring the importance of phosphorylation-dependent signaling in the regulation of epithelial maturation and structural integrity.

Collectively, this work delineates the structural and transcriptional foundations governing intestinal epithelial maturation in rainbow trout, deciphering the genetic programming that coordinates barrier formation and functional specialization. Beyond providing unprecedented insight into salmonid intestinal biology, these findings establish a foundational framework for developing targeted interventions to enhance intestinal health. This model serves as a preclinical in vitro platform for evaluating the impact of nutritional regimes, pathogen challenges, and novel aquaculture conditions on epithelial integrity, repair mechanisms, and overall barrier function.

## Supplementary Information

**Additional file 1: Evaluation of epithelial architecture and nuclear overlap at 28 dps. A. **Representative confocal Z-stack of mature epithelium (28 dps) rendered with depth-coding to facilitate visualization ofnuclear overlap and estimation of stratification. Image was processed using NIS-Viewer software (version 4.20, b972). **B. **Three-dimensional reconstruction of nuclei (DAPI, blue) generated from a confocal Z-stack using Imaris software (Bitplane, v7.7) to further visualize nuclear overlap and assess multilayer formation.

**Additional file 2:** **Apical structures. A.** Distribution of rosette-shaped apical structures. On the left, a representative scanning electron microscopy image of the epithelium at 14 dps. **B.** SEM images of rosette-shaped apical structures observed throughout epithelial maturation at 1, 7-, 14-, 21-, and 28 dps are shown. Light-blue arrows indicate these structures (8000 x magnification) (n= 3).

**Additional file 3:** Table containing the list of differentially expressed genes (DEGs) between mature and immature epithelium and enrichment functions based on Gene Ontology and KEGG for up-regulated and down-regulated genes.

## Data Availability

The datasets used and/or analyzed during the current study are available from the corresponding author on reasonable request.
