# Supplementary material for: From immature to mature epithelium: unveiling structural dynamics and transcriptional programs in rainbow trout intestinal barrier
Source: Vet Res. 2026 Sep 15;57:165. doi: 10.1186/s13567-026-01766-2 (PMC13576363; doi:10.1186/s13567-026-01766-2)
Supplement: Supplementary file 2 [file 13567_2026_1766_MOESM2_ESM.pdf]

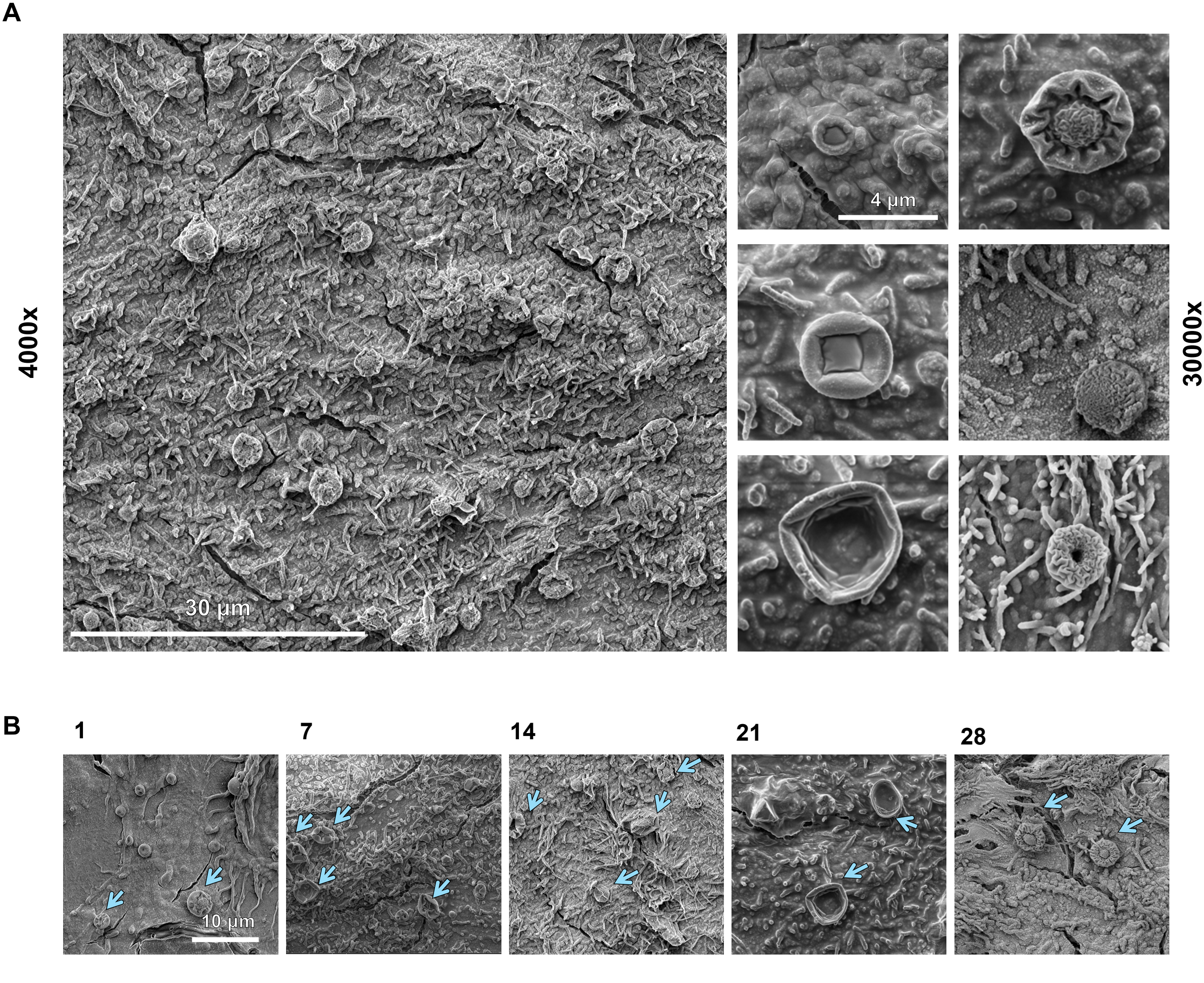
A
4000x
30 μm
4 μm
30000x
B
1
7
14
21
28
10 μm
